# Supplementary material for: Above- and below-ground trait coordination in tree seedlings depend on the most limiting resource: a test comparing a wet and a dry tropical forest in Mexico
Source: PeerJ. 2022 Jun 14;10:e13458. doi: 10.7717/peerj.13458 (PMC9205306; doi:10.7717/peerj.13458)
Supplement: Supplemental Information 1 [file peerj-10-13458-s001.docx]

## Supplemental information

### Above and below ground trait coordination in tree seedlings depend on the most limiting resource: A test comparing a wet and a dry tropical forest in Mexico

### Plant material

#### Tropical rain forest

During the rainy season, we searched for seedlings from 42 tree and 1 liana species that had no senescent cotyledons and at least one pair of leaves (average 21 seedlings per species, range 4-60). We extracted the seedlings from the soil extremely carefully by excavating a hole with a diameter of three times the height of the seedling and as deep as needed to get the tip of the principal root, to preserve the integrity of all root tissues (following Paz 2003). Seedlings were kept in moist plastic bags inside a cooler and transported to a field laboratory for processing.

#### Tropical dry forest

Within the boundaries of Chamela Biological Station, we collected seeds from at least 10 individuals of each of 28 species during the peak of fruit production. Seeds were placed in wet sand beds in a greenhouse for germination. Fifteen days after the radicle emerged, when the first pair of leaves was fully expanded, we randomly chose 16 seedlings per species. We measured biomass at the beginning of the experiment for 8 seedlings and the rest were transplanted into 5 L pots (16 cm diameter and 30 cm tall) with basal drainage (one seedling per pot) containing local forest soil. The pot position was assigned in a randomized block design to statistically control for solar radiation and temperature variation in the greenhouse. Plants were grown at water saturation. After 3 months, seedlings were carefully extracted to preserve the integrity of all root tissues and within a few hours, transported to laboratory for further processing, following Pineda-García et al. (2011).

**Literature cited**

Arsenault, J. L., S. Poulcur, C. Messier, and R. Guay. 1995. WinRHlZO^TM^, a Root-measuring System with a Unique Overlap Correction Method. HortScience 30:906–906.

Paz, H. 2003. Root/Shoot Allocation and Root Architecture in Seedlings: Variation among Forest Sites, Microhabitats, and Ecological Groups. Biotropica 35:318–332.

Pineda-García, F., H. Paz, and C. Tinoco-Ojanguren. 2011. Morphological and physiological differentiation of seedlings between dry and wet habitats in a tropical dry forest: Water-use strategies of TDF tree seedlings. Plant, Cell & Environment 34:1536–1547.

Rasband, W. S. 2014. ImageJ. Java 1.6.0_20 (64-bit), National Institutes of Health, Bethesda, Maryland, USA.

van Breugel, M., F. Bongers, and M. Martínez-Ramos. 2007. Species Dynamics During Early Secondary Forest Succession: Recruitment, Mortality and Species Turnover. Biotropica 39:610–619.
