# Supplementary material for: Above- and below-ground trait coordination in tree seedlings depend on the most limiting resource: a test comparing a wet and a dry tropical forest in Mexico
Source: PeerJ. 2022 Jun 14;10:e13458. doi: 10.7717/peerj.13458 (PMC9205306; doi:10.7717/peerj.13458)
Supplement: Supplemental Information 2 [file peerj-10-13458-s002.docx]

|  | **Moist forest** | **Dry forest** | **References** |
| --- | --- | --- | --- |
| - Natural protected area | Chajul | Chamela |  |
| - Forest type | Tropical moist forest | Tropical dry forest |  |
| - Topography | Low to high hills | Low to high hills | Cotler et al. 2002, Ibarra-Manríquez and Martínez-Ramos 2002 |
| - Average annual precipitation | 3,000 mm | 748 mm | Cotler et al. 2002, Ibarra-Manríquez and Martínez-Ramos 2002 |
| - Average annual temperature | 22 | 24.9 | Cotler et al. 2002, Ibarra-Manríquez and Martínez-Ramos 2002 |
| - Understorey transmittance (%) wet season | 1.14 – 2.53  1.83 (1.46) | 2.00 – 20.00  13.7 (11.01) | Matsuo et al. 2021, Parker et al. 2005 |
| - Understorey transmittance (%) dry season |  | 36.20 – 87.70 -  51.20 (15.71) | Parker et al. 2005 |
| - Dry season (months) | 3 | 6 – 8 | Cotler et al. 2002, Ibarra-Manríquez and Martínez-Ramos 2002 |
| - Soil | Humic acrisol | Poorly developed soils, Regosols, Lixisols, Cambisols, and  Phaeozems | Gavito et al. 2018, Ibarra-Manríquez and Martínez-Ramos 2002 |
| - Soil average available nitrogen (mg kg^-1^) | 12.2 – 57.9 | 10.0 – 84.1 | Ibarra-Manríquez and Martínez-Ramos 2002, Cuevas-Reyes *et al.* 2004 |
| - Soil average available phosphorous (mg kg^-1^) | 0.10 – 0.95 | 0.19 – 1.70 | Ibarra-Manríquez and Martínez-Ramos 2002, Cuevas-Reyes *et al.* 2004 |
