# Supplementary material for: Above- and below-ground trait coordination in tree seedlings depend on the most limiting resource: a test comparing a wet and a dry tropical forest in Mexico
Source: PeerJ. 2022 Jun 14;10:e13458. doi: 10.7717/peerj.13458 (PMC9205306; doi:10.7717/peerj.13458)
Supplement: Supplemental Information 3 [file peerj-10-13458-s003.docx]

|  |  |  |  | **FUNCTIONAL TRAITS MEASURED** | | | | | | | | | | |
| --- | --- | --- | --- | --- | --- | --- | --- | --- | --- | --- | --- | --- | --- | --- |
| **FOREST** | **CODE** | **SPECIES** | **FAMILY** | **LTh** | **WD** | **SLA** | **LDMC** | **LWC** | **MPU** | **SWC** | **RTh** | **RD** | **SRL** | **MRD** |
| Moist | Aca-hay | *Acacia hayesii* | Fabaceae | x | x | x | x | x | x | x | x | x | x | x |
| Moist | Aca-sp | *Acacia sp* | Fabaceae | x | x | x | x | x | x | x | x | x | x | x |
| Moist | Alc-lat | *Alchornea latifolia* | Euphorbiaceae | x | x | x | x | x | x | x | x | x | x | x |
| Moist | Bel-sp | *Belluccia sp* | Melastomataceae | x | x | x | x | x | x | x | x | x | x | x |
| Moist | Bro-ali | *Brosimum alicastrum* | Moraceae | x | x | x | x | x | x | x | x | x | x | x |
| Moist | Bro-cos | *Brosimum costaricanum* | Moraceae | x | x | x | x | x | x | x | x | x | x | x |
| Moist | Bro-gui | *Brosimum guianense* | Moraceae | x | x | x | x | x | x | x | x | x | x | x |
| Moist | Bur-sim | *Bursera simaruba* | Burseraceae | x | x | x | x | x | x | x | x | x | x | x |
| Moist | Cal-bra | *Calophyllum brasiliense* | Clusiaceae | x | x | x | x | x | x | x | x | x | x | x |
| Moist | Cas-ela | *Castilla elastica* | Moraceae | x | x | x | x | x | x | x | x | x | x | x |
| Moist | Cec-pel | *Cecropia peltata* | Urticaceae | x | x | x | x | x | x | x | x | x | x | x |
| Moist | Cor-all | *Cordia alliodora* | Boraginaceae | x | x | x | x | x | x | x | x | x | x | x |
| Moist | Cro-sch | *Croton schiedeanus* | Euphorbiaceae | x | x | x | x | x | x | x | x | x | x | x |
| Moist | Cup-gla | *Cupania glabra* | Sapindaceae | x | x | x | x | x | x | x | x | x | x | x |
| Moist | Cym-sp | *Cymbopetalum sp* | Annonaceae | x | x | x | x | x | x | x | x | x | x | x |
| Moist | Dal-gla | *Dalbergia glabra* | Fabaceae | x | x | x | x | x | x | x | x | x | x | x |
| Moist | Dia-gui | *Dialium guianense* | Fabaceae | x | x | x | x | x | x | x | x | x | x | x |
| Moist | Gua-gla | *Guarea glabra* | Meliaceae | x | x | x | x | x | x | x | x | x | x | x |
| Moist | Ing-pav | *Inga pavoniana* | Fabaceae | x | x | x | x | x | x | x | x | x | x | x |
| Moist | Lic-hyp | *Licania hypoleuca* | Chrysobalanaceae | x | x | x | x | x | x | x | x | x | x | x |
| Moist | Lon-cru | *Lonchocarpus sericeus* | Fabaceae | x | x | x | x | x | x | x | x | x | x | x |
| Moist | Lys-aca | *Lysiloma acapulcense* | Fabaceae | x | x | x | x | x | x | x | x | x | x | x |
| Moist | Och-pyr | *Ochroma pyramidale* | Malvaceae | x | x | x | x | x | x | x | x | x | x | x |
| Moist | Ore-pel | *Oreopanax peltatus* | Araliaceae | x | x | x | x | x | x | x | x | x | x | x |
| Moist | Pou-sp | *Pouteria sp* | Sapotaceae | x | x | x | x | x | x | x | x | x | x | x |
| Moist | Psy-cus | *Psychotria cuspidata* | Rubiaceae | x | x | x | x | x | x | x | x | x | x | x |
| Moist | Psy-mic | *Psychotria micrantha* | Rubiaceae | x | x | x | x | x | x | x | x | x | x | x |
| Moist | Psy-sp1 | *Psychotria sp1* | Rubiaceae | x | x | x | x | x | x | x | x | x | x | x |
| Moist | Psy-sp2 | *Psychotria sp2* | Rubiaceae | x | x | x | x | x | x | x | x | x | x | x |
| Moist | Pte-roh | *Pterocarpus rohrii* | Fabaceae | x | x | x | x | x | x | x | x | x | x | x |
| Moist | Sch-par | *Schizolobium parahyba* | Fabaceae | x | x | x | x | x | x | x | x | x | x | x |
| Moist | Sip-the | *Siparuna thecaphora* | Monimiaceae | x | x | x | x | x | x | x | x | x | x | x |
| Moist | Spo-mom | *Spondias mombin* | Anacardiaceae | x | x | x | x | x | x | x | x | x | x | x |
| Moist | Tab-alb | *Tabernaemontana alba* | Apocynaceae | x | x | x | x | x | x | x | x | x | x | x |
| Moist | The-aho | *Thevetia ahouai* | Apocynaceae | x | x | x | x | x | x | x | x | x | x | x |
| Moist | Tre-mic | *Trema micrantha* | Cannabaceae | x | x | x | x | x | x | x | x | x | x | x |
| Moist | Tri-ery | *Trichilia erythrocarpa* | Meliaceae | x | x | x | x | x | x | x | x | x | x | x |
| Moist | Tri-gal | *Trichospermum galeottii* | Malvaceae | x | x | x | x | x | x | x | x | x | x | x |
| Moist | Tro-rac | *Trophis racemosa* | Moraceae | x | x | x | x | x | x | x | x | x | x | x |
| Moist | Vat-lun | *Vatairea lundellii* | Fabaceae | x | x | x | x | x | x | x | x | x | x | x |
| Moist | Voc-gua | *Vochysia guatemalensis* | Vochysiaceae | x | x | x | x | x | x | x | x | x | x | x |
| Moist | Xyl-fru | *Xylopia frutescens* | Annonaceae | x | x | x | x | x | x | x | x | x | x | x |
| Moist | Zan-jun | *Zanthoxylum juniperinum* | Rutaceae | x | x | x | x | x | x | x | x | x | x | x |
| Dry | Aca-far | *Acacia farnesiana* | Fabaceae | x | x | x | x | x | x | x | x | x | x | x |
| Dry | Aca-sp | *Acacia sp* | Fabaceae | x | x | x | x | x | x | x | x | x | x | x |
| Dry | Amp-ads | *Amphipterygium adstringens* | Anacardiaceae | x | x | x | x | x | x | x | x | x | x | x |
| Dry | Apo-pan | *Apoplanesia paniculata* | Fabaceae | x | x | x | x | x | x | x | x | x | x | x |
| Dry | Ast-gra | *Astronium graveolens* | Anacardiaceae | x | x | x | x | x | x | x | x | x | x | x |
| Dry | Bau-pau | *Bauhinia pauletia* | Fabaceae | x | x | x | x | x | x | x |  |  |  | x |
| Dry | Bur-fag | *Bursera fagaroides* | Burseraceae | x | x | x | x | x | x | x |  |  |  | x |
| Dry | Bur-ins | *Bursera instabilis* | Burseraceae | x | x | x | x | x | x | x |  |  |  | x |
| Dry | Bur-sim | *Bursera simaruba* | Burseraceae | x | x | x | x | x | x | x | x | x | x | x |
| Dry | Cae-cal | *Caesalpinia caladenia* | Fabaceae | x | x | x | x | x | x | x | x | x | x | x |
| Dry | Cae-cor | *Caesalpinia coriaria* | Fabaceae | x | x | x | x | x | x | x |  |  |  | x |
| Dry | Cae-eri | *Caesalpinia eriostachys* | Fabaceae | x | x | x | x | x | x | x | x | x | x | x |
| Dry | Cae-pla | *Caesalpinia platyloba* | Fabaceae | x | x | x | x | x | x | x | x | x | x | x |
| Dry | Cei-aes | *Ceiba aesculifolia* | Malvaceae | x | x | x | x | x | x | x |  |  |  | x |
| Dry | Coc-bar | *Coccoloba barbadensis* | Polygonaceae | x | x | x | x | x | x | x |  |  |  | x |
| Dry | Coc-lie | *Coccoloba liebmannii* | Polygonaceae | x | x | x | x | x | x | x |  |  |  | x |
| Dry | Coc-vit | *Cochlospermum vitifolium* | Bixaceae | x | x | x | x | x | x | x | x | x | x | x |
| Dry | Cor-alb | *Cordia alba* | Boraginaceae | x | x | x | x | x | x | x |  |  |  | x |
| Dry | Cor-all | *Cordia alliodora* | Boraginaceae | x | x | x | x | x | x | x |  |  |  | x |
| Dry | Cor-ela | *Cordia elaeagnoides* | Boraginaceae | x | x | x | x | x | x | x | x | x | x | x |
| Dry | Cro-ala | *Croton alamosanus* | Euphorbiaceae | x | x | x | x | x | x | x |  |  |  | x |
| Dry | Cro-pse | *Croton pseudoniveus* | Euphorbiaceae | x | x | x | x | x | x | x |  |  |  | x |
| Dry | Cro-sub | *Croton suberosus* | Zygophyllaceae | x | x | x | x | x | x | x |  |  |  | x |
| Dry | Ent-cyc | *Enterolobium cyclocarpum* | Fabaceae | x | x | x | x | x | x | x |  |  |  | x |
| Dry | Ery-lan | *Erythrina lanceolata* | Fabaceae | x | x | x | x | x | x | x | x | x | x | x |
| Dry | Gli-sep | *Gliricidia sepium* | Fabaceae | x | x | x | x | x | x | x | x | x | x | x |
| Dry | Gua-cou | *Guaiacum coulteri* | Zygophyllaceae | x | x | x | x | x | x | x |  |  |  | x |
| Dry | Gua-ulm | *Guazuma ulmifolia* | Malvaceae | x | x | x | x | x | x | x | x | x | x | x |
| Dry | Hae-bra | *Haematoxylum brasiletto* | Fabaceae | x | x | x | x | x | x | x |  |  |  | x |
| Dry | Han-chr | *Handroanthus chrysanthus* | Bignoniaceae | x | x | x | x | x | x | x | x | x | x | x |
| Dry | Hel-pal | *Heliocarpus pallidus* | Malvaceae | x | x | x | x | x | x | x | x | x | x | x |
| Dry | Hin-lat | *Hintonia latiflora* | Rubiaceae | x | x | x | x | x | x | x | x | x | x | x |
| Dry | Jat-cha | *Jatropha chamelensis* | Euphorbiaceae | x | x | x | x | x | x | x | x | x | x | x |
| Dry | Jat-mal | *Jatropha malacophylla* | Euphorbiaceae | x | x | x | x | x | x | x |  |  |  | x |
| Dry | Jat-sta | *Jatropha standleyi* | Euphorbiaceae | x | x | x | x | x | x | x | x | x | x | x |
| Dry | Lon-con | *Lonchocarpus constrictus* | Fabaceae | x | x | x | x | x | x | x |  |  |  | x |
| Dry | Lon-eri | *Lonchocarpus eriocarinalis* | Fabaceae | x | x | x | x | x | x | x | x | x | x | x |
| Dry | Lon-mag | *Lonchocarpus magallanesii* | Fabaceae | x | x | x | x | x | x | x | x | x | x | x |
| Dry | Lys-mic | *Lysiloma microphylla* | Fabaceae | x | x | x | x | x | x | x | x | x | x | x |
| Dry | Mim-aca | *Mimosa acantholoba* | Fabaceae | x | x | x | x | x | x | x |  |  |  | x |
| Dry | Mim-are | *Mimosa arenosa* | Fabaceae | x | x | x | x | x | x | x | x | x | x | x |
| Dry | Pip-obl | *Piptadenia obliqua* | Fabaceae | x | x | x | x | x | x | x | x | x | x | x |
| Dry | Plu-rub | *Plumeria rubra* | Apocynaceae | x | x | x | x | x | x | x | x | x | x | x |
| Dry | Rau-tet | *Rauvolfia tetraphylla* | Apocynaceae | x | x | x | x | x | x | x |  |  |  |  |
| Dry | Rec-mex | *Recchia mexicana* | Surianaceae | x | x | x | x | x | x | x | x | x | x | x |
| Dry | Rup-pal | *Ruprechtia pallida* | Polygonaceae | x | x | x | x | x | x | x | x | x | x | x |
| Dry | Sen-ato | *Senna atomaria* | Fabaceae |  | x | x | x | x |  |  |  |  |  | x |
| Dry | Sen-sp | *Senna sp* | Fabaceae | x | x | x | x | x | x | x |  |  |  | x |
| Dry | Spo-pur | *Spondias purpurea* | Anacardiaceae | x | x | x | x | x | x | x |  |  |  | x |
| Dry | Swi-hum | *Swietenia humilis* | Meliaceae | x | x | x | x | x | x | x | x | x | x | x |
| Dry | Tab-ros | *Tabebuia rosea* | Bignoniaceae | x | x | x | x | x | x | x |  |  |  | x |
| Dry | Zap-For | *Zapoteca Formosa* | Fabaceae | x | x | x | x | x | x | x |  |  |  | x |
