# Supplementary material for: Above- and below-ground trait coordination in tree seedlings depend on the most limiting resource: a test comparing a wet and a dry tropical forest in Mexico
Source: PeerJ. 2022 Jun 14;10:e13458. doi: 10.7717/peerj.13458 (PMC9205306; doi:10.7717/peerj.13458)
Supplement: Supplemental Information 4 — SLA (specific leaf area); MPU (minimum photosynthetic unit); LTh (leaf thickness); LWC (leaf water content ); WD (wood density); SWC (stem water content); SRL (specific root length ); MRD (maximum root depth); RTh (root thickness); RD (root density). [file peerj-10-13458-s004.docx]

| **Species means** |  | **Moist forest** | | **Dry forest** | |
| --- | --- | --- | --- | --- | --- |
|  | **Traits** | **PC1** | **PC2** | **PC1** | **PC2** |
|  | % Variance explained | 35.31 | 18.23 | 46.57 | 14.61 |
| Leaf | SLA | -0.25 | 0.02 | -0.03 | 0.51 |
|  | LTh | -0.26 | -0.46 | 0.31 | -0.33 |
|  | LWC | -0.43 | -0.10 | 0.42 | 0.04 |
|  | MPU | -0.25 | -0.43 | 0.30 | -0.41 |
| Stem | WD | 0.43 | -0.11 | -0.37 | -0.02 |
|  | SWC | -0.44 | 0.18 | 0.42 | -0.02 |
| Root | SRL | -0.32 | -0.06 | 0.20 | 0.48 |
|  | RTh | 0.03 | 0.54 | 0.28 | 0.39 |
|  | RD | 0.30 | -0.49 | -0.27 | -0.25 |
|  | MRD | 0.21 | -0.12 | -0.36 | 0.14 |
| **PICs** |  | **Moist forest** | | **Dry forest** |  |
|  | **Traits** | **PC1** | **PC2** | **PC1** | **PC2** |
|  | % Variance explained | 37.06 | 18.83 | 37.87 | 21.49 |
| Leaf | SLA | 0.17 | 0.15 | 0.12 | -0.61 |
|  | LTh | 0.23 | 0.38 | 0.26 | 0.43 |
|  | LWC | 0.41 | 0.20 | 0.46 | 0.01 |
|  | MPU | 0.29 | 0.26 | 0.16 | 0.59 |
| Stem | WD | -0.44 | 0.19 | -0.34 | 0.27 |
|  | SWC | 0.44 | -0.13 | 0.44 | -0.07 |
| Root | SRL | 0.33 | 0.22 | 0.27 | -0.08 |
|  | RTh | 0.05 | -0.65 | 0.32 | -0.01 |
|  | RD | -0.35 | 0.45 | -0.29 | -0.11 |
|  | MRD | -0.22 | 0.04 | -0.33 | 0.06 |
