# Supplementary material for: Above- and below-ground trait coordination in tree seedlings depend on the most limiting resource: a test comparing a wet and a dry tropical forest in Mexico
Source: PeerJ. 2022 Jun 14;10:e13458. doi: 10.7717/peerj.13458 (PMC9205306; doi:10.7717/peerj.13458)
Supplement: Supplemental Information 5 — Pearson coefficient (r) and sample size (n) are shown. Values are ordered from the lowest to the highest p-value. SLA (specific leaf area); MPU (minimum photosynthetic unit); LTh (leaf thickness); LWC (leaf water content); WD (wood density); SWC (stem water content); SRL (specific root length); MRD (maximum root depth); RTh (root thickness); RD (root density). [file peerj-10-13458-s005.docx]

|  |  | | **Moist** | | **forest** | | **Dry** | | **forest** | | |  | |  |
| --- | --- | --- | --- | --- | --- | --- | --- | --- | --- | --- | --- | --- | --- | --- |
| **Trait 1** | **Trait 2** | | **r** | | **n** | | **r** | | **n** | | | **z** | | **p** |
| SRL | | RTh | | -0.50 | | 43 | | 0.55 | | 28 | 4.61 | | 0.000 | |
| LWC | | RTh | | -0.12 | | 43 | | 0.54 | | 28 | 2.82 | | 0.005 | |
| WD | | MRD | | 0.15 | | 43 | | 0.63 | | 51 | 2.80 | | 0.005 | |
| SWC | | MRD | | -0.18 | | 43 | | -0.66 | | 50 | 2.79 | | 0.005 | |
| SLA | | RTh | | -0.25 | | 43 | | 0.23 | | 28 | 1.94 | | 0.053 | |
| SLA | | SWC | | 0.39 | | 43 | | 0.01 | | 51 | 1.90 | | 0.058 | |
| MPU | | RTh | | -0.12 | | 43 | | 0.31 | | 28 | 1.71 | | 0.087 | |
| LTh | | MPU | | 0.73 | | 43 | | 0.51 | | 51 | 1.69 | | 0.091 | |
| LTh | | RTh | | -0.18 | | 43 | | 0.22 | | 28 | 1.55 | | 0.121 | |
| MPU | | MRD | | -0.05 | | 43 | | -0.33 | | 50 | 1.37 | | 0.172 | |
| LTh | | SWC | | 0.19 | | 43 | | 0.45 | | 51 | 1.37 | | 0.172 | |
| SLA | | SRL | | 0.38 | | 43 | | 0.05 | | 28 | 1.36 | | 0.173 | |
| MPU | | SWC | | 0.23 | | 43 | | 0.47 | | 51 | 1.27 | | 0.205 | |
| SWC | | RTh | | 0.10 | | 43 | | 0.39 | | 28 | 1.23 | | 0.218 | |
| LTh | | MRD | | -0.07 | | 43 | | -0.29 | | 50 | 1.09 | | 0.277 | |
| SLA | | RD | | -0.08 | | 43 | | 0.19 | | 28 | 1.08 | | 0.280 | |
| LTh | | RD | | -0.01 | | 43 | | -0.27 | | 28 | 1.08 | | 0.282 | |
| SRL | | RD | | -0.45 | | 43 | | -0.64 | | 28 | 1.04 | | 0.300 | |
| MPU | | RD | | 0.01 | | 43 | | -0.24 | | 28 | 1.01 | | 0.312 | |
| RTh | | MRD | | -0.02 | | 43 | | -0.26 | | 28 | 0.97 | | 0.334 | |
| LWC | | MPU | | 0.37 | | 43 | | 0.51 | | 51 | 0.86 | | 0.390 | |
| WD | | RTh | | -0.15 | | 43 | | -0.32 | | 28 | 0.72 | | 0.472 | |
| WD | | RD | | 0.47 | | 43 | | 0.32 | | 28 | 0.71 | | 0.477 | |
| LWC | | SWC | | 0.60 | | 43 | | 0.50 | | 51 | 0.69 | | 0.488 | |
| LTh | | WD | | -0.35 | | 43 | | -0.47 | | 51 | 0.66 | | 0.507 | |
| SLA | | MPU | | -0.10 | | 43 | | 0.04 | | 51 | 0.65 | | 0.515 | |
| LWC | | MRD | | -0.39 | | 43 | | -0.50 | | 51 | 0.62 | | 0.537 | |
| SWC | | RD | | -0.50 | | 43 | | -0.37 | | 28 | 0.60 | | 0.546 | |
| SRL | | MRD | | -0.24 | | 43 | | -0.09 | | 28 | 0.60 | | 0.550 | |
| LWC | | RD | | -0.30 | | 43 | | -0.42 | | 28 | 0.54 | | 0.587 | |
| SLA | | LTh | | -0.04 | | 43 | | -0.15 | | 51 | 0.53 | | 0.595 | |
| MPU | | SRL | | 0.09 | | 43 | | -0.03 | | 28 | 0.49 | | 0.625 | |
| MPU | | WD | | -0.39 | | 43 | | -0.47 | | 51 | 0.45 | | 0.654 | |
| WD | | SRL | | -0.28 | | 43 | | -0.18 | | 28 | 0.44 | | 0.660 | |
| SLA | | MRD | | -0.12 | | 43 | | -0.21 | | 51 | 0.41 | | 0.682 | |
| SWC | | SRL | | 0.38 | | 43 | | 0.29 | | 28 | 0.39 | | 0.695 | |
| LWC | | WD | | -0.49 | | 43 | | -0.54 | | 52 | 0.28 | | 0.778 | |
| SLA | | WD | | -0.28 | | 43 | | -0.25 | | 52 | 0.15 | | 0.878 | |
| WD | | SWC | | -0.82 | | 43 | | -0.83 | | 51 | 0.14 | | 0.891 | |
| SLA | | LWC | | 0.45 | | 43 | | 0.43 | | 52 | 0.11 | | 0.913 | |
| RTh | | RD | | -0.50 | | 43 | | -0.52 | | 28 | 0.08 | | 0.932 | |
| LTh | | LWC | | 0.43 | | 43 | | 0.42 | | 51 | 0.05 | | 0.962 | |
| LTh | | SRL | | 0.12 | | 43 | | 0.13 | | 28 | 0.04 | | 0.971 | |
| LWC | | SRL | | 0.37 | | 43 | | 0.37 | | 28 | 0.00 | | 0.998 | |
| RD | | MRD | | 0.29 | | 43 | | 0.29 | | 28 | 0.00 | | 0.998 | |
