# Supplementary figures and images for: Above- and below-ground trait coordination in tree seedlings depend on the most limiting resource: a test comparing a wet and a dry tropical forest in Mexico
Source: PeerJ. 2022 Jun 14;10:e13458. doi: 10.7717/peerj.13458 (PMC9205306; doi:10.7717/peerj.13458)

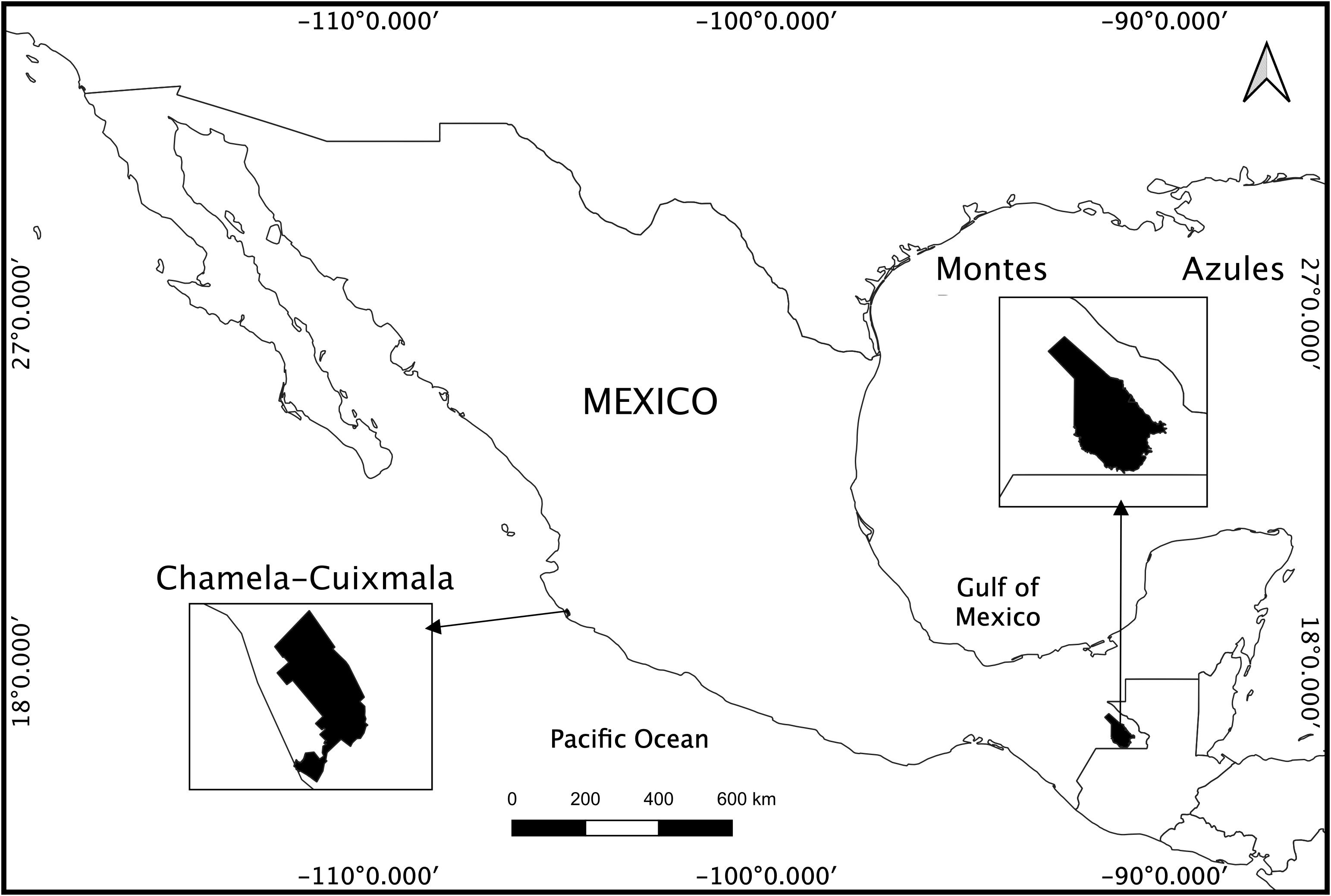

Supplement: Supplemental Information 6 [file peerj-10-13458-s006.jpg]

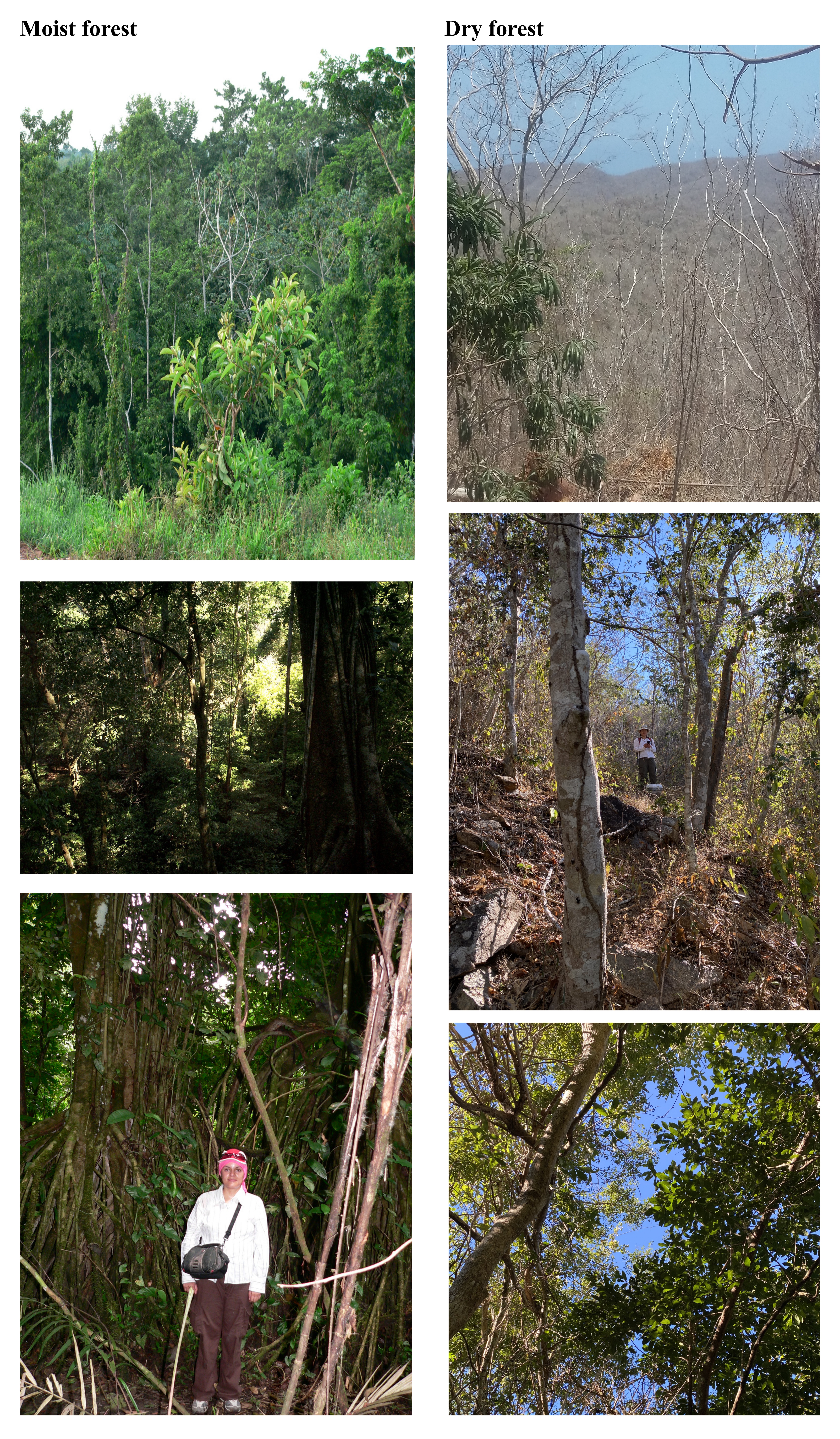

Supplement: Supplemental Information 7 [file peerj-10-13458-s007.jpg]

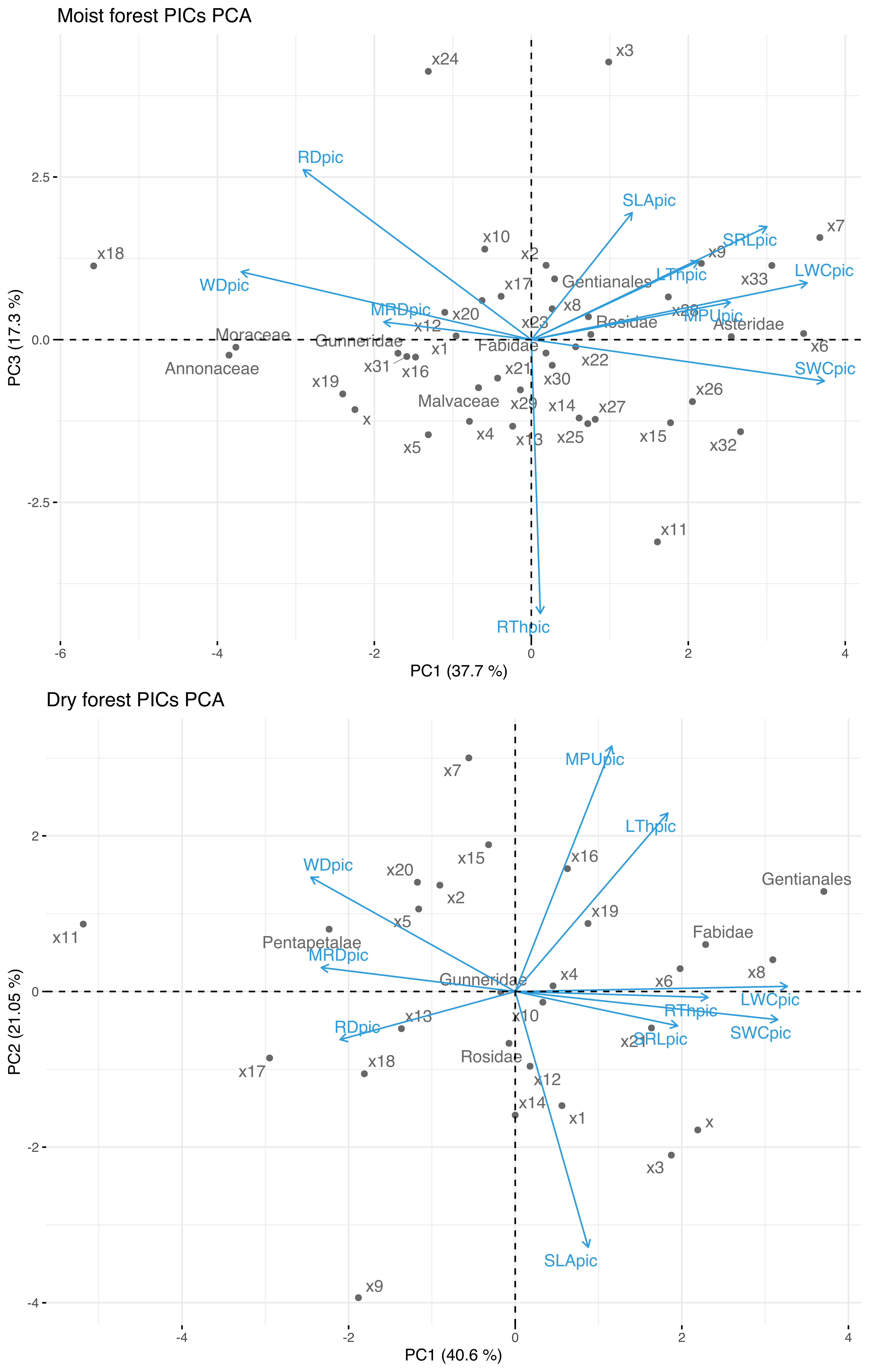

Supplement: Supplemental Information 8 — SLA (specific leaf area); MPU (minimum photosynthetic unit); LTh (leaf thickness); LWC (leaf water content); WD (wood density); SWC (Stem water content ); SRL (specific root length); MRD (maximum root depth); RTh (root thickness); RD (root density). The suffix pic indicate phylogenetic independent contrast. [file peerj-10-13458-s008.png]

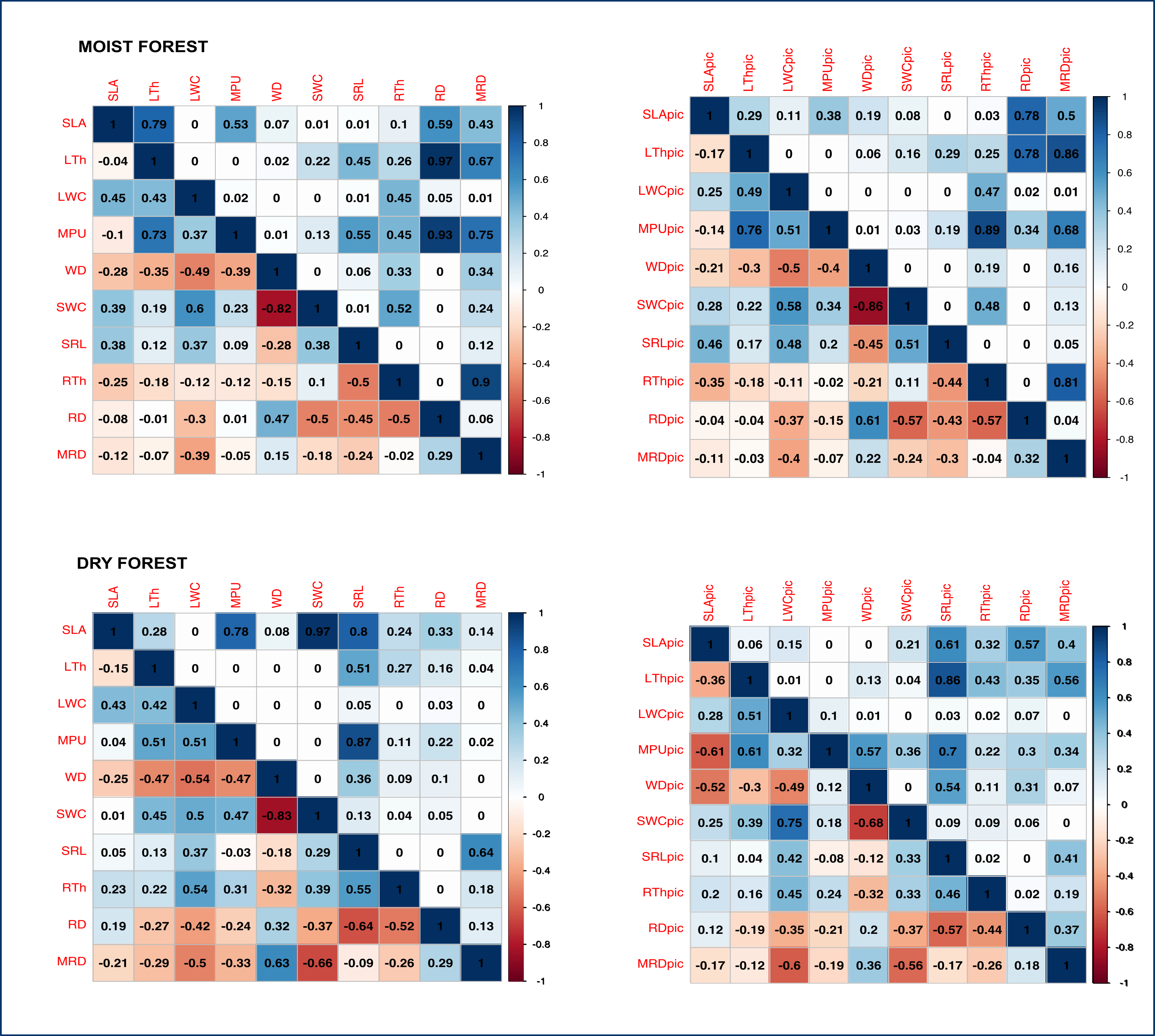

Supplement: Supplemental Information 9 — In each matrix, the lower triangle corresponds to correlation coefficients (r), and the upper triangle to p-values. Colors indicate the strength and sign of correlation (blue positive, red, negative). [file peerj-10-13458-s009.png]
